# Intestinal FXR deficiency uncouples steatosis protection from liver inflammation and fibrosis in MASH-diet fed mice

**DOI:** 10.1038/s41598-026-60976-y

**Published:** 2026-07-10

**Authors:** Margaux Nawrot, Simon Peschard, Morgane M Thibaut, Kévin Ory, Emmanuelle Vallez, Emilie Dorchies, Véronique Touche, Julie Dubois-Chevalier, Céline Gheeraert, Sandrine Caron, Kadiombo Bantubungi, Philippe Lefebvre, Anne Tailleux, Joel T Haas, Laure B Bindels, Isabelle Leclercq, David Dombrowicz, Sophie Lestavel, Bart Staels

**Affiliations:** 1 Univ. Lille, CHU Lille, Institut Pasteur de Lille, U1011-EGID, Inserm, Lille, F- 59000 France; 2 Metabolism and Nutrition Research Group, Louvain Drug Research Institute, Université catholique de Louvain, UCLouvain, Brussels, Belgium; 3 Welbio Department, WEL Research Institute, Wavre, Belgium; 4 Laboratory of Hepato-Gastroenterology, Institute of Experimental and Clinical Research, Université Catholique de Louvain, Brussels, Belgium; 5 Lille University, INSERM, CHU de Lille, U1011-EGID, Institut Pasteur de Lille, 1 rue du Professeur Calmette 59019, Lille, France; 6 Université Lyon1, INSERM, INRAE, CarMeN laboratory, Hôpital Lyon Sud, Secteur 2, Bâtiment Cens Eli-2D, 165 chemin du Grand Revoyet, Oullins-Pierre-Bénite, 69310 France

**Keywords:** FXR, MASH, Intestine-liver crosstalk, Liver steatosis protection, Liver inflammation, Diseases, Gastroenterology, Immunology, Microbiology

## Abstract

The Farnesoid X Receptor (FXR), a nuclear bile acid (BA) receptor highly expressed in the liver and intestine, is a potential pharmacological target for Metabolic dysfunction-Associated SteatoHepatitis (MASH). While intestinal FXR inhibition reduces high-fat diet (HFD)-induced hepatic steatosis, its role in MASH progression remains unclear. This study investigates the impact of intestinal FXR-deficiency on MASH development in a diet-induced murine model. Intestinal FXR-deficient (^int^FXR KO) and control mice were fed a high-fat, sucrose, and cholesterol-enriched diet (HFSC) for 24 weeks. Intestinal immune phenotyping, microarray, 16 S rRNA sequencing, bile acid quantification and liver assessments (histology, biochemistry and single-cell RNA sequencing (scRNA-seq)) were performed. ^int^FXR KO mice were protected against HFSC diet-induced obesity and hepatic steatosis but exhibited altered expression of intestinal barrier-associated genes, with increased cytotoxic CD8^+^ T-lymphocytes. Microbiota composition and bile acid profiles were altered, including reduced Lachnospiraceae species correlating negatively with liver hyocholic acid levels. Despite a protection against hepatic steatosis, liver inflammation and fibrosis were unchanged in ^int^FXR KO mice. Transcriptomic and Immune cell scRNA-seq analysis revealed alteration in immune-related pathways with an increased neutrophil proportion and higher cDC1:cDC2 and CD4:CD8 T cell ratios. Thus, intestinal FXR-deficiency limits steatosis but promote a distinct hepatic immune-inflammatory response and does not prevent progression to MASH.

**Supplementary Information:**

The online version contains supplementary material available at https://doi.org/10.1038/s41598-026-60976-y.

## Introduction

Metabolic dysfunction-associated steatotic liver disease (MASLD) is a progressive hepatic disease displaying a phenotypic spectrum progressing from isolated steatosis (MASL) to steatohepatitis (MASH) in which steatosis, inflammation and hepatocyte ballooning are simultaneously present. The molecular mechanisms involved in MASL to MASH transition are still incompletely understood^1,2^. MASH patients display an increased risk to develop liver fibrosis leading to cirrhosis and hepatocarcinoma^3^.

Bile acids (BA) are amphipathic molecules produced by the liver from cholesterol as primary BA, mainly conjugated to taurine in mice and to glycine in humans, secreted into the intestine after a meal, where they act as detergents to facilitate intestinal lipid absorption. In the digestive tract, BA are modified by the microbiota to secondary BA and then reabsorbed in the distal intestine to be recycled by the liver via the portal vein, the enterohepatic cycle^4^. In addition to their detergent properties, BA also act as signaling molecules, targeting specific proteins such as the nuclear receptor Farnesoid X Receptor (FXR). In liver and intestine, FXR controls numerous homeostatic pathways such as BA synthesis, lipid and carbohydrate metabolism^4^. Several FXR agonists have been evaluated for treatment of MASH. Notably, the semi-synthetic BA obeticholic acid (OCA) improves hepatic histological characteristics in patients with MASH^5–7^. However, results from the REGENERATE trial on MASH reported mixed results with an improvement in liver fibrosis, but not in MASH resolution in OCA-treated patients, indicating complex actions of FXR in MASLD^6^.

Indeed, pharmacological FXR activation with ligands such as OCA does not allow to distinguish the selective effects of liver vs. intestinal FXR activation. Given the accumulating evidence on the importance of the intestine-liver crosstalk in the control of energy homeostasis^8^, studies using tissue-specific pharmacological agents and/or tissue-specific modulation of FXR activity have been undertaken. In mice, activation of FXR with the intestinal-restricted compound fexaramine reduced body weight gain, hepatic glucose production and altered the BA pool, with a decrease of plasma primary BA and an increase of secondary BA such as lithocholic acid^9^. Moreover, fexaramine treatment increased energy expenditure by promoting beiging of adipose tissue, thereby protecting mice from High Fat Diet (HFD)-induced obesity^10^. Fexaramine treatment also improved the enterohepatic BA cycle, lipid metabolism and reduced ethanol-induced liver disease in mice^11^. This specific intestinal FXR agonist also improves gut barrier function in Dextran Sulfate Sodium (DSS)-treated mice^12^. The second generation fexaramine-like agonist LH10 reduces histological features of MASH such as steatosis, inflammation foci and serum alanine aminotransferase (ALT) levels^13^. On the other hand, MASH patients treated with aldafermin, a Fibroblast Growth Factor 19 (FGF19) analog and major intestinal FXR target gene showed no improvement in liver fibrosis despite a significant reduction in fatty liver and an improvement in markers of hepatic cytolysis compared to patients on placebo^14^.

Thus, whether FXR should be activated or deactivated in the treatment of metabolic diseases remains controversial. Indeed, whole-body FXR knock-out (KO) mice are protected against HFD-induced obesity and insulin resistance^15^. Intestine-specific FXR deficient mice display reduced hepatic steatosis associated with a lower body weight gain and less hepatic triglyceride (TG) accumulation upon HFD feeding^16,17^. Surprisingly, an increase in hepatic TG levels was also reported in “fast food” fed ^int^FXR KO mic^18^. Moreover, FXR antagonism also protects against HFD-induced obesity, insulin resistance and liver steatosis^15–17,19,20^. Furthermore, de-activation of intestinal FXR upon BA sequestrant treatment improves lipid and glucose homeostasis by lowering LDL-cholesterol, decreasing intestinal glucose absorption, promoting splanchnic glucose utilization and increasing Glucagon-Like Peptide 1 (GLP-1) secretion^21–23^. However, while most of these studies use metabolic dysfunction models, only limited data are available regarding the response of ^int^FXR KO mice to specific MASH-inducing diets. We hypothesized that the protection against hepatic steatosis conferred by intestinal FXR deficiency could prevent the development of hepatic inflammation and fibrosis under MASH-inducing conditions.

To address this question, we investigated whether protection against obesity and hepatic steatosis conferred by intestinal FXR deficiency is accompanied by protection against inflammatory and fibrotic liver remodeling during MASH progression. To this end, ^int^FXR KO mice were challenged with a diet-induced MASH model recapitulating key metabolic and hepatic features of human MASLD in the setting of obesity and insulin resistance^24^.

## Results

### Impact of intestinal epithelium-specific FXR-deficiency on body weight gain and intestinal transcriptome in HFSC diet fed mice

Compared to Ctrl-flox littermate mice, FXR mRNA and protein are decreased to undetectable levels in all parts of the intestine in chow-diet fed ^int^FXR KO mice. By contrast, no change in FXR expression was observed in liver and kidney (Fig. S1a; *p* = 0.0043; Fig. S1c, *p* = 0.026). The absence of functional FXR in ^int^FXR KO mice is illustrated by a decreased expression of its target genes *Fgf15* and *Shp* in both ileum (*p* = 0.0079) and colon (*p* = 0.0286 and *p* = 0.0381, respectively) (Fig. S1d). Moreover, the decreased intestinal FGF15 in ^int^FXR KO mice is associated with an increase in hepatic *Cyp7a1* expression (Fi.. S1e, *p* = 0.0159). By contrast, liver FXR activity is not modified in ^int^FXR KO mice, as *Shp* mRNA was similar in the livers of the two genotypes (Fig. S1e).

**Fig. 1:**
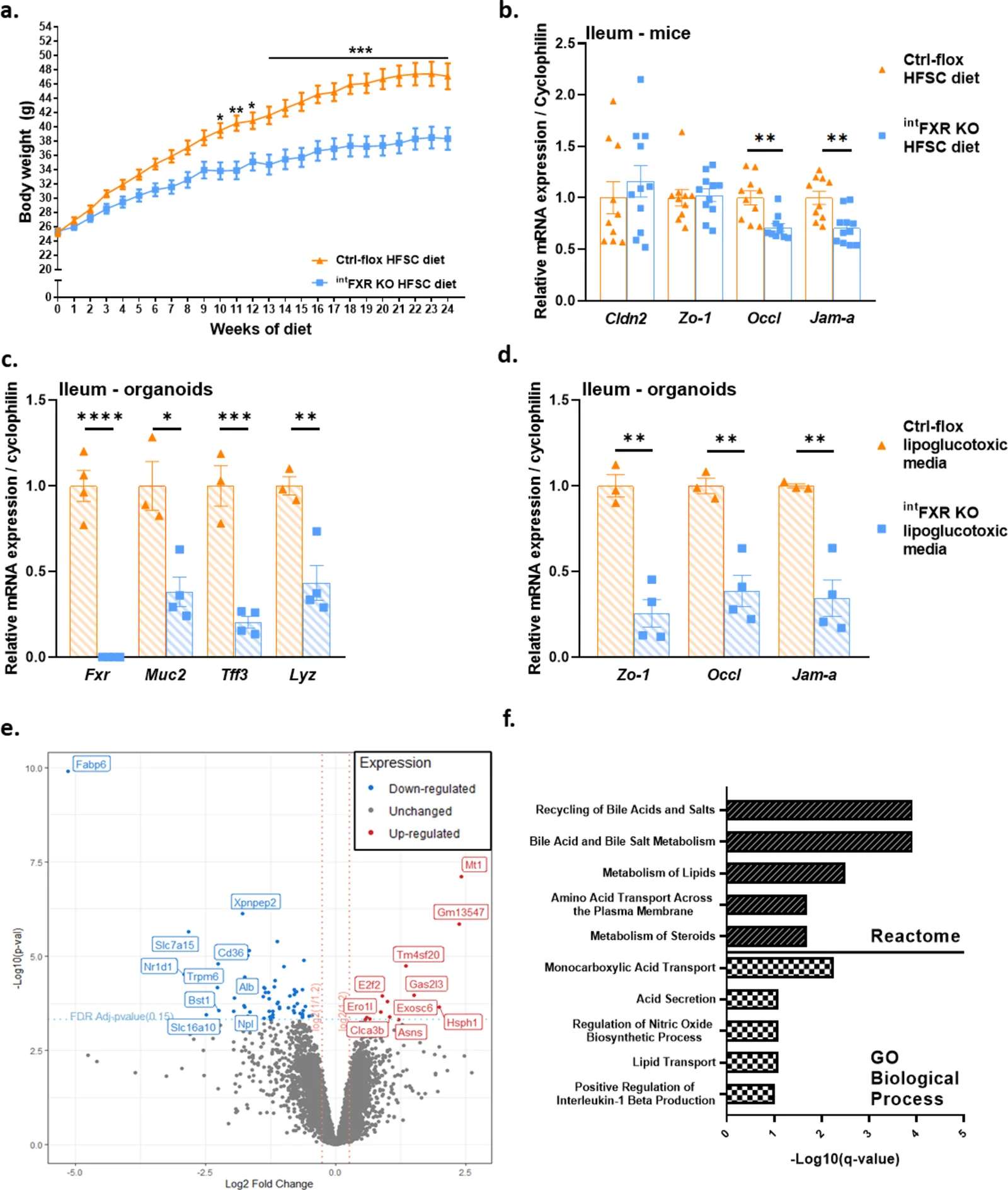
Effect of intestinal FXR deficiency in mice and intestinal organiods under MASH-inducing conditions: body weight evolution, barrier-associated gene expression, and ileal transcriptomic profile. **(a)** Body weight evolution of Control (Ctrl-flox) and ^int^FXR KO mice (n = 12/genotype) fed a HFSC diet for 24 weeks. **(b)** qPCR quantification of selected tight junction-related mRNA expression in whole ileal tissue from Ctrl-flox and ^int^FXR KO mice (n = 12/genotype) after 24 weeks of HFSC diet. **(c)** qPCR quantification of epithelial cell marker genes and **(d)** of selected tight junction-related genes in ileum organoids derived from Ctrl-flox and ^int^FXR KO mice cultured under lipoglucotoxic conditions (n = 3-4/genotype). **(e)** Volcano plot showing differentially expressed genes in ileal tissue from Ctrl-flox versus ^int^FXR KO mice after 24 weeks of HFSC diet (n = 5/genotype). **(f)** Pathway enrichment analysis of differentially expressed ileal genes identified by microarray analysis. Data are presented as mean +/- SEM. Statistical analysis: **(a)** Two-way ANOVA followed by Sidak’s multiple comparison test. Time effect: *p < 0.001*; genotype effect: ** *p < 0.01* and *** *p < 0.001.* **(b)**, Mann-Whitney test: **p < 0.01. **(c & d)** Unpaired-student’s t test: * *p < 0.05*, **p < 0.01, ***p < 0.001 and **** *p < 0.0001*. **(e & f)** Microarray analysis performed using Galaxy software and GIANT application. Differential expression analysis was performed using LIMMA with FDR correction (adjusted p-value < 0.15; fold change threshold +/- 1.2). (Abbreviation: FC, Fold Change; FDR, false discovery rate; HFSC diet, High-fat diet enriched with sucrose and cholesterol).

Under chow diet, both genotypes exhibited similar body weight (Fig. S1f). However, ^int^FXR KO mice were protected against HFSC diet-induced weight gain (Fig. 1a; *p* = 0.0160 at 10 weeks of diet and *p* < 0.0001 at 24 weeks of diet). After HFSC diet feeding, ^int^FXR KO mice had an average body weight of 38 vs. 47 g for the Ctrl-flox littermate mice (Fig. 1a, *p* < 0.001). Intestinal *Fgf15*,* Shp* and *Fabp6* mRNA levels remained reduced after diet feeding (Fig. S1g, *p* < 0.001 for *Shp* and *Fabp6*; *p* = 0.0083 for *Fgf15*).

Since whole-body FXR deficiency alters the intestinal barrier^25^, we measured the expression levels of markers of intestinal barrier function. The mRNA levels of tight junction-associated proteins such as *Occludin* and *Jam-a* was decreased in HFSC-fed ^int^FXR KO mice (*p* = 0.002 and *p* = 0.003, respectively), whereas *Claudin-2* nor *Zo-1* were altered (Fig. 1b). Twenty-four weeks of HFSC diet feeding also resulted in reduced mRNA levels in ^int^FXR KO mice of *Muc2* and *Lyz1* (*p* = 0.004 and *p* = 0.0109, respectively), markers of Goblet and Paneth cells respectively (Fi. S1h). Despite the decreased expression of these markers of intestinal barrier function in ^int^FXR KO mice, macromolecule absorption, as assessed by the FITC-dextran assay, did not reveal any differences between the two genotypes (data not shown), indicating no massive alteration in intestinal permeability. Interestingly, intestinal barrier dysregulation was also observed in ileal ^int^FXR KO organoids cultured under lipoglucotoxic conditions with reduced expression of genes associated to mucus-producing cells (*Muc2* and *Tff3*) (*p* = 0.011 and *p* = 0.0007, respectively), Paneth cells (*Lyz1*) (*p* = 0.007) and tight junctions (*Zo-1*, *p* = 0.0011; *Occludin*, *p* = 0.0031 and *Jam-a*, *p* = 0.0035) (Fig. 1c&d).

Unbiased analysis was next performed to determine the transcriptomic differences between the ilea of 24 weeks HFSC diet-fed ^int^FXR KO and Ctrl-flox mice. Seventy-six differentially expressed genes were identified between the two genotypes, displaying a minimal fold change of 1.2 and adj-p value < 0.15, required for downstream analysis (Fig. 1e & Table S1). These include direct FXR target genes such as *Fabp6*,* Slc51a* (or *Ostα*) and *Slc51b* (or *Ostβ*) (Fig. 1e & Table S1). The differentially expressed genes are part of pathways such as absorption of nutrients, BA metabolism and, more intriguingly, immune regulation, such as the positive regulation of Il-1β production pathway (Fig. 1f).

### Impact of intestinal epithelium-specific FXR deficiency on BA metabolism and intestinal immunity in HFSC diet fed mice

Since FXR plays a key role in intestinal BA re-uptake and regulates liver BA synthesis *via* FGF-15 ^26^, we next assessed potential modifications of the BA pool by analyzing the BA composition in caecal content and livers of ^int^FXR KO compared to control littermate mice after 24 weeks of HFSC diet feeding.

**Fig. 2:**
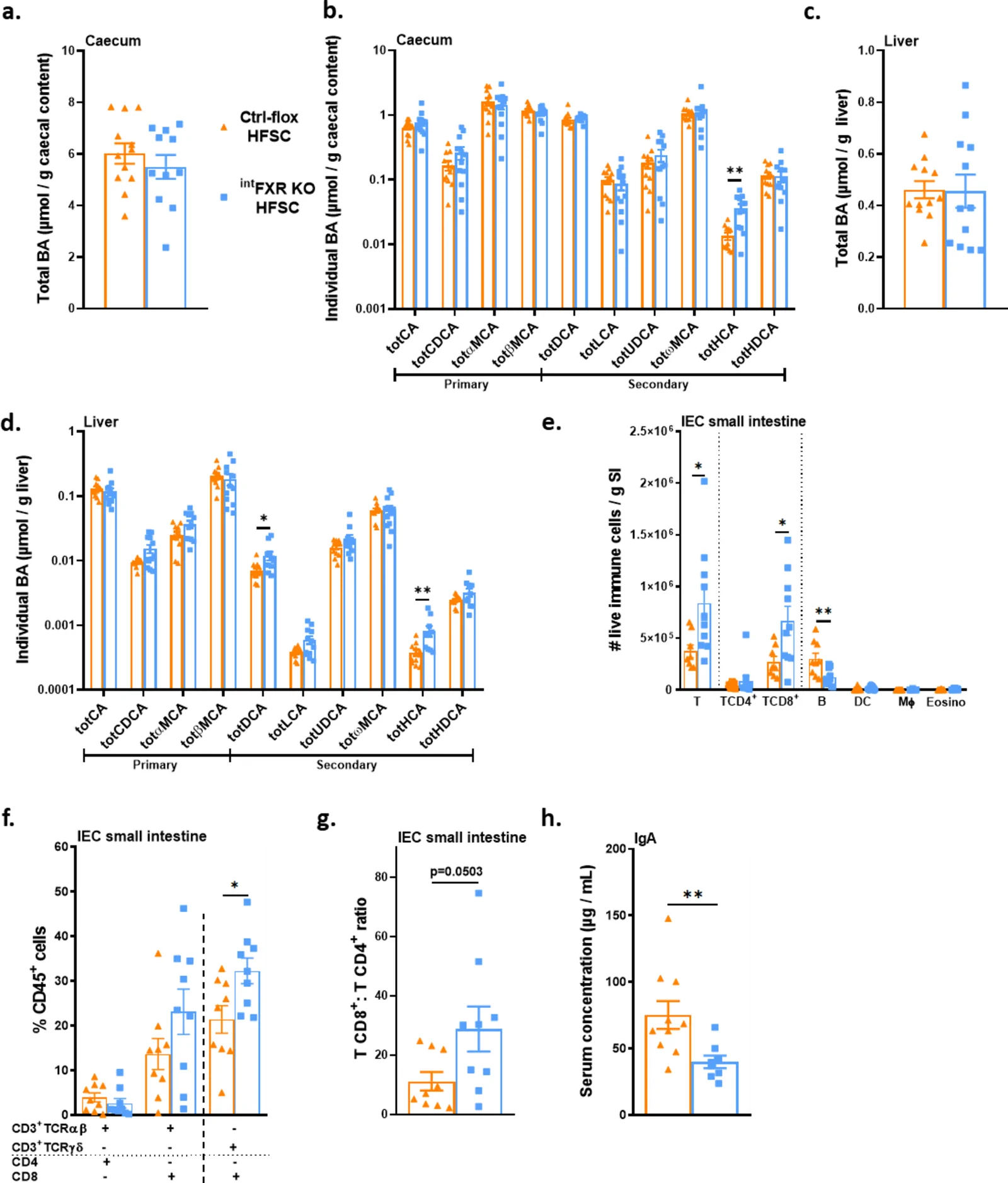
Effect of intestinal FXR deficiency on BA composition and intestinal immune cell proportions after 24 weeks of HFSC diet. **(a)** Total bile acid quantification and **(b)** relative bile acid composition in caecal content from Ctrl-flox and ^int^FXR KO mice after 24 weeks of HFSC feeding (*n* = 11–12/genotype). **(c)** Total bile acid quantification and **(d)** relative bile acid composition in liver from Ctrl-flox and ^int^FXR KO mice after 24 weeks of HFSC feeding (*n* = 11–12/genotype). **(e)** Quantification of intestinal immune cells population and **(f)** quantification of intestinal T lymphocyte subpopulations isolated from small intestinal epithelium by flow cytometry from Ctrl-flox and ^int^FXR KO mice after 24 weeks of HFSC feeding (*n* = 9/genotype). **(g)** Intestinal epithelial T CD8^+^: T CD4^+^ ratio and **(h)** Serum IgA concentrations from Ctrl-flox and ^int^FXR KO mice after 24 weeks of HFSC feeding (*n* = 8–10/genotype). Ctrl-flox mice are represented by orange triangles and ^int^FXR KO mice by blue squares. Data are presented as mean +/- SEM. Statistical analysis.: Mann-Whitney, *p < 0.05 and **p < 0.01. (Abbreviation: B, B lymphocytes; CA, Cholic acid; CDCA, Chenodeoxycholic acid; DC, Dendritic cells; DCA, Deoxycholic acid; Eosino, eosinophils; HCA, Hyocholic acid; HDCA, Hyodeoxycholic acid; HFSC, High-fat diet enriched with sucrose and cholesterol; IEC, Intra-epithelial cells; IgA, Immunoglobulin A; isoDCA, Isodeoxycholic Acid; LCA, Lithocholic acid; MΦ, macrophages; SI, Small intestine; T, T lymphocytes; UDCA, Ursodeoxycholic acid; αMCA, α-Muricholic acid; βMCA, β-Muricholic acid; ωMCA, ω-Muricholic acid).

Despite the expected blunted *Fxr* and *Fgf15* expression and the modification of BA-related transcriptional pathways in the ileum (Fig. 1f & Fig. S1g), caecal total BA concentrations were similar in both genotypes (Fig. 2A). Interestingly, only total hyocholic acid (HCA) (free and conjugated) was significantly higher in the caecum of ^int^FXR KO vs. Ctrl-flox mice (Fig. 2b, *p* = 0.0036).

In the liver, BA concentrations were similar in HFSC-fed ^int^FXR KO compared to Ctrl-flox mice (Fig. 2c), with small but significant increases in total DCA (0.0119 µmol/g liver for ^int^FXR KO mice vs. 0.0071 µmol/g in control littermate mice, *p* = 0.0129) and total HCA (0.008 µmol/g vs. 0.004 µmol/g in control littermates, *p* = 0.0048) concentrations (Fig. 2d). Since these BA changes were not pronounced, we further investigated the second pathway identified by our transcriptomics analysis, the regulation of immunity.

Characterization of the immune cell populations by flow cytometry (Figure S2a) revealed similar immune cell populations in the intestinal epithelium and in the *lamina propria* of ^int^FXR KO and control mice upon chow diet feeding (Fig. S2d&e). Both genotypes displayed no difference in intraepithelial CD3^+^ T lymphocyte (T) proportions (Fig. S2b), but ^int^FXR KO mice displayed a higher proportion of TCRαβ^+^CD8^+^ T cells (*p* = 0.0071) and a lower proportion of TCRγδ^+^CD8^+^ T cells (*p* = 0.0426) (Fig.S2c). Upon feeding the HFSC diet the immune cell populations in the *lamina propria* remained similar when comparing the two genotypes (Fig. S2f&g). Interestingly, ^int^FXR KO mice displayed significantly higher numbers of intra-epithelial CD3^+^ T cells than the Ctrl-flox mice (8.41 × 10^5^ cells/g of tissue vs. 3.74 × 10^5^ cells/g in ctrl-flox mice, *p* = 0.016) (Fig. 2e). This is mainly due to a significant increase in cytotoxic CD8^+^ T cells in the intestinal epithelium of ^int^FXR KO mice (6.69 × 10^5^ cells/g vs. 2.71 × 10^5^ cells/g, *p* = 0.043) (Fig. 2e). Qualitative analysis of the intraepithelial CD8^+^ T population of the two genotypes on HFSC diet showed higher TCRγδ^+^CD8^+^ T (> 32% vs. 21%; *p* = 0.04) (Fig. 2f). This increase in the number of CD8^+^ T cells is confirmed by an upward trend in the T CD8^+^: T CD4^+^ ratio in the intestinal epithelium of ^int^FXR KO mice (Fig. 2g, *p* = 0.0503). Thus, on HFSC diet feeding, intestinal epithelium FXR deficiency leads to an increase of intraepithelial TCRγδ^+^CD8^+^ T cells in the gut epithelium.

Finally, the B lymphocyte numbers were lower in the intestinal epithelium of ^int^FXR KO mice either fed a chow or a HFSC diet (Fig. 2e, *p* = 0.0057 and Figure S2b, *p* = 0.0044) and serum levels of immunoglobulin A (IgA), involved in epithelial protection^27^, were significantly lower in HFSC diet fed ^int^FXR KO vs. Ctrl-flox mice (-53%; *p* = 0.006) (Fig. 2h).

### Impact of intestinal epithelial-specific FXR deficiency on the gut microbiota in HFSC diet fed mice

**Fig. 3:**
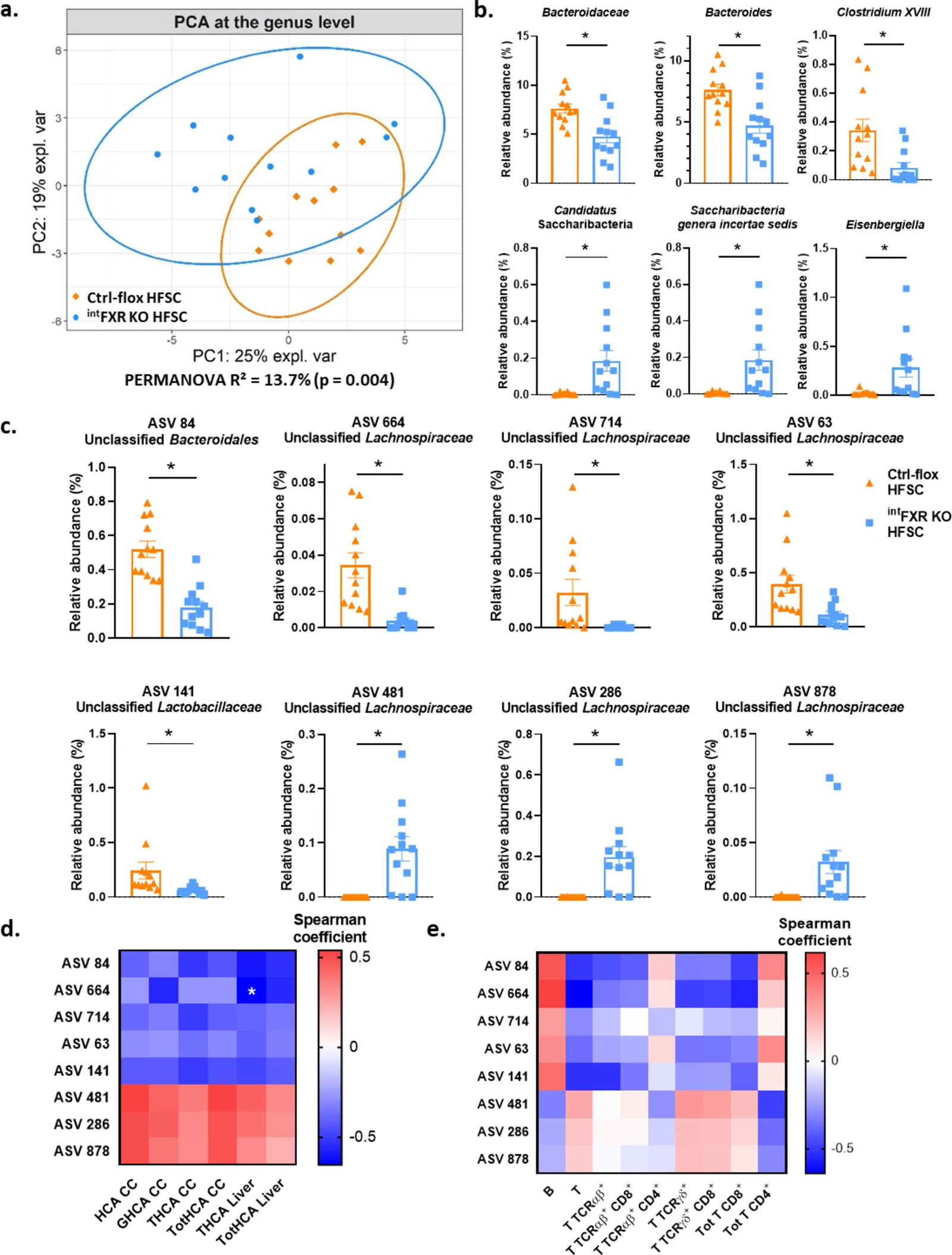
Effect of intestinal FXR deficiency on caecal microbiota composition after 24 weeks of HFSC diet. **(a)** Principal component analysis (PCA) based on bacteria genera abundance in caecal content from Ctrl-flox and ^int^FXR KO mice after 24 weeks of HFSC diet (*n* = 12/genotype). **(b)** Relative abundance of bacterial taxa at family and genus levels and **(c)** Differential abundance analysis of Amplicon Sequence Variants (ASVs) identified in caecal content from Ctrl-flox and ^int^FXR KO mice after 24 weeks of HFSC diet (*n* = 12/genotype). Heatmap showing Spearman correlations between ASV abundance and **(d)** liver and caecal bile acid concentrations and **(e)** immune cell populations isolated from small intestinal epithelium. For PCA analysis **(a)**, group differences were assessed using PERMANOVA (R² = 13.7%, *p* = 0.004). For taxonomic and ASV analyses **(b & c)**, data are presented as mean +/- SEM and statistical significance was assessed using Mann–Whitney U-tests. For correlation analyses **(d & e)**, Spearman’s rank correlation tests were performed. P-values were adjusted for multiple testing using the Benjamini–Hochberg false discovery rate (FDR) procedure. *q-value < 0.05. (Abbreviation: BL, B lymphocytes; CC, Caecal content; GHCA, Glycohyocholic acid; HCA, Hyocholic acid; HFSC, High-fat diet enriched with sucrose and cholesterol; TCR, T cell receptor; TL, T lymphocytes).

Since the intestinal microbiota play an important role in liver and intestinal homeostasis, we compared the caecal microbiota composition by 16 S rRNA gene sequencing in ^int^FXR KO and Ctrl-flox mice after 24 weeks of HFSC diet feeding. Principal component analysis (PCA) at the genus level revealed a heterogeneity in the microbiota composition between ^int^FXR KO and Ctrl-flox mice, with 13.7% of the variance being explained by genotype (Fig. 3a, *p* = 0.004). The relative abundance of 2 families and 4 genera were different (*q-value < 0.05*): the relative abundance of *Bacteroides* (and Bacteroidaceae family) and *Clostridium XVII* was lower in ^int^FXR KO caecal content, while *Saccharibacteria Genera Incertae Sedis* (and *Candidatus* Saccharibacteria family) and *Eisenbergiella* were higher (from nearly 0% to respectively 0.18% and 0.28% of total microbial populations) (Fig. 3b). Amplicon Sequencing Variants (ASV) analysis identified changes in the relative abundance of 8 ASV between ^int^FXR KO and Ctrl-flox mice (Fig. 3c; *q-value* < 0.05). In line, the lower *Bacteroidaceae* in ^int^FXR KO was related to the decrease of ASV 84 identified as *Bacteriodales*. Moreover, subtle changes in the *Lachnospiraceae* family were observed. Indeed, ASV 664, ASV 714 and ASV 63 were significantly lower in caecal contents of ^int^FXR KO compared to littermate controls, while ASV 481, ASV 286 and ASV 878 were higher (Fig. 3c).

As BA and gut immunity are strongly linked to the microbiota, we performed correlation analysis of all these data. The increased *Lachnospiraceae* tended to positively correlate with free, glyco-conjugated and total HCA caecal concentrations (Fig. 3d). In addition, the lower abundance of ASV 84, ASV 664 and ASV 141 in ^int^FXR KO mice (Fig. 3c) correlated negatively with the hepatic concentration of tauro-conjugated HCA (THCA) and total HCA (Fig. 3d). Of note, ASV 664, a *Lachnospiraceae* less abundant in HFSC diet fed ^int^FXR KO mice (Fig. 3c), correlated positively with the B cells (Fig. 3e). Furthermore, ASV 664 correlated negatively with T cell numbers, especially TCRγδ^+^CD8^+^ T cells (Fig. 3e). Finally, ASV 84 and ASV141 also showed positive correlations with B cell and negative correlations with T cell numbers (Fig. 3e).

Altogether, the immune cell changes observed in ^int^FXR KO mice were only modestly associated with bile acid and intestinal microbiota profiles. While several bacterial species correlated with intestinal immune markers, these observations remain associative and do not establish causality. Further mechanistic studies will be required to determine whether microbiota alterations contribute to the observed intestinal immune phenotype.

### Impact of intestinal epithelium-specific FXR deficiency on liver steatosis in HFSC diet fed mice

**Fig. 4:**
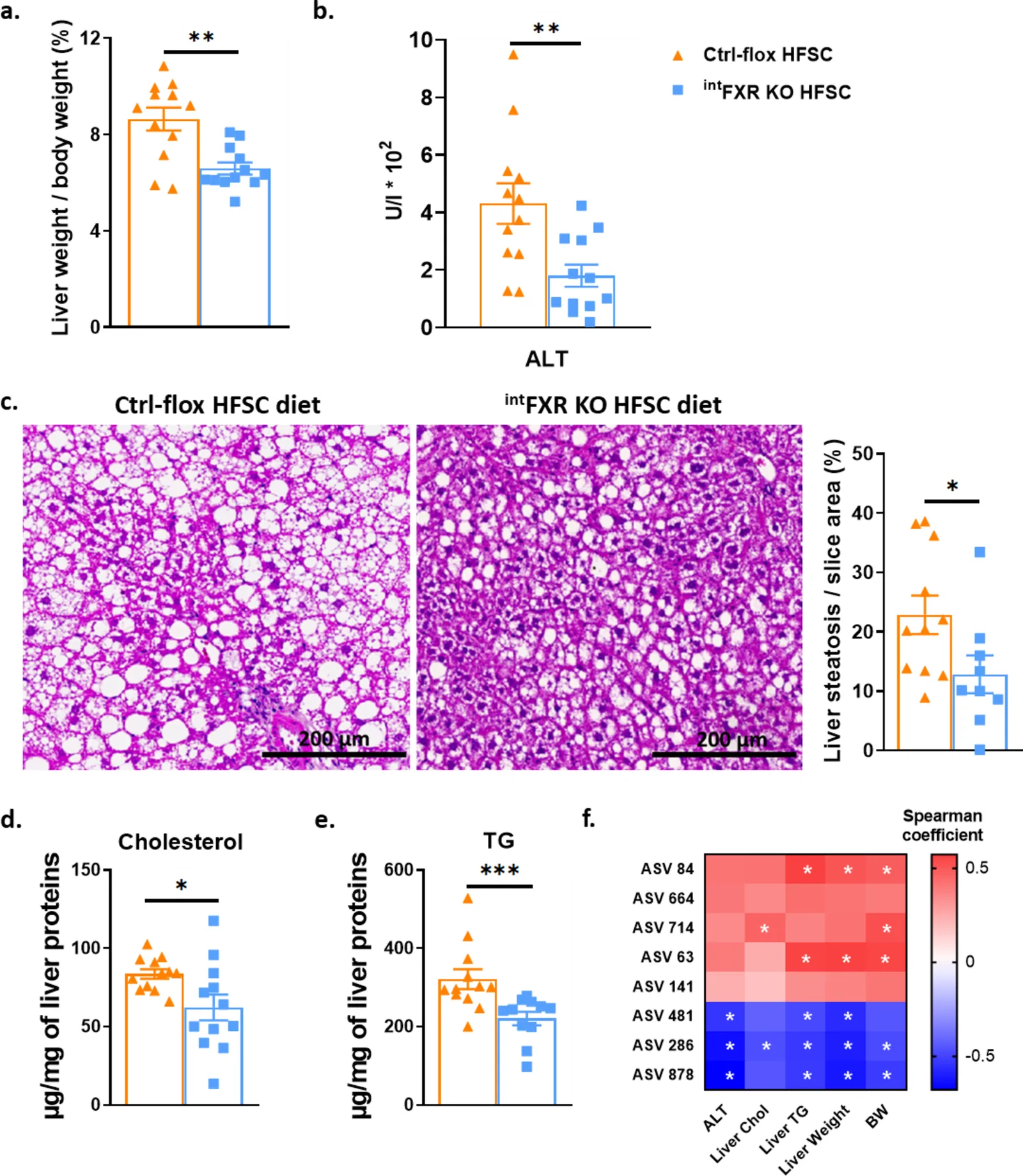
Effect of intestinal FXR deficiency on liver weight, hepatic steatosis and liver lipid accumulation after 24 weeks of HFSC diet. **(a)** Liver to body weight ratio measured at sacrifice and **(b)** circulating ALT concentrations in Ctrl-flox and ^int^FXR KO mice after 24 weeks of HFSC diet (*n* = 12/genotype). **(c)** Representative hematoxylin and eosin (H&E)-stained liver sections and quantification of steatotic area normalized to total liver area excluding vascular lumen in Ctrl-flox and ^int^FXR KO mice after 24 weeks of HFSC diet (*n* = 9–11/genotype). **(d)** Hepatic cholesterol concentrations and **(e)** hepatic TG concentrations normalized to total hepatic protein content in Ctrl-flox and ^int^FXR KO mice after 24 weeks of HFSC diet (*n* = 11–12/genotype). **(f)** Heatmap showing Spearman correlations between ASV abundance, body weight, and hepatic parameters. For **(a–e)**, data are presented as mean +/- SEM and statistical significance was assessed using Mann–Whitney tests. *p < 0.05, **p < 0.01, ***p < 0.001. For **(f)**, Spearman’s rank correlation tests were performed and p-values were adjusted for multiple testing using the Benjamini–Hochberg false discovery rate (FDR) procedure. * *q-value < 0.05*. (Abbreviations: ALT, Alanine aminotransferase; BW, Body weight; Chol, Cholesterol; H&E, Hematoxylin and eosin; HFSC diet, High-fat diet enriched with sucrose and cholesterol; TG, Triglycerides)

We next assessed the impact of HFSC diet feeding on liver health of ^int^FXR KO mice. Chow diet-fed ^int^FXR KO mice showed no difference in liver weight, nor hepatic concentrations of triglycerides and cholesterol compared to littermate control mice (Fig. S3a-d). After 24 weeks of HFSC diet feeding, ^int^FXR KO mice showed a lower liver weight (6.6% of body weight compared to 8.6% for control mice, *p* = 0.007) (Fig. 4a). Plasma ALT levels were significantly lower in ^int^FXR KO mice (180 U/l compared to 431 U/l in Ctrl-flox mice; *p* = 0.004) suggesting a protection from hepatic cytolysis (Fig. 4b). Moreover, histological analysis showed that livers from ^int^FXR KO mice displayed less steatosis (13%, compared to 23% in livers from Ctrl-flox mice, *p* = 0.025) (Fig. 4c), associated with lower hepatic concentrations of cholesterol and triglycerides (62 µg/mg and 221 µg/mg protein respectively in ^int^FXR KO mice, compared to 84 µg/mg and 322 µg/mg in Ctrl-flox mice, *p* = 0.025 for cholesterol, *p* < 0.001 for TG) (Fig. 4d&e).

We then assessed whether changes in hepatic parameters in ^int^FXR KO mice correlate with microbiota alterations. ASV 481, ASV 286 and ASV 878 showed negative correlations with hepatic metabolic parameters such as circulating ALT and AST levels and liver TG concentration, as well as body and liver weights (Fig. 4f). Other ASVs, in particular ASV 84 and ASV 63, showed a positive correlation with TG concentrations, liver weight and body weight (Fig. 4f). Although any causal association cannot be deducted from associations, these data show that microbiota changes in ^int^FXR KO mice correlate with parameters related to hepatic lipid metabolism.

### Impact of intestinal epithelium-specific FXR deficiency on liver inflammation and fibrosis in HFSC diet fed mice

**Fig. 5:**
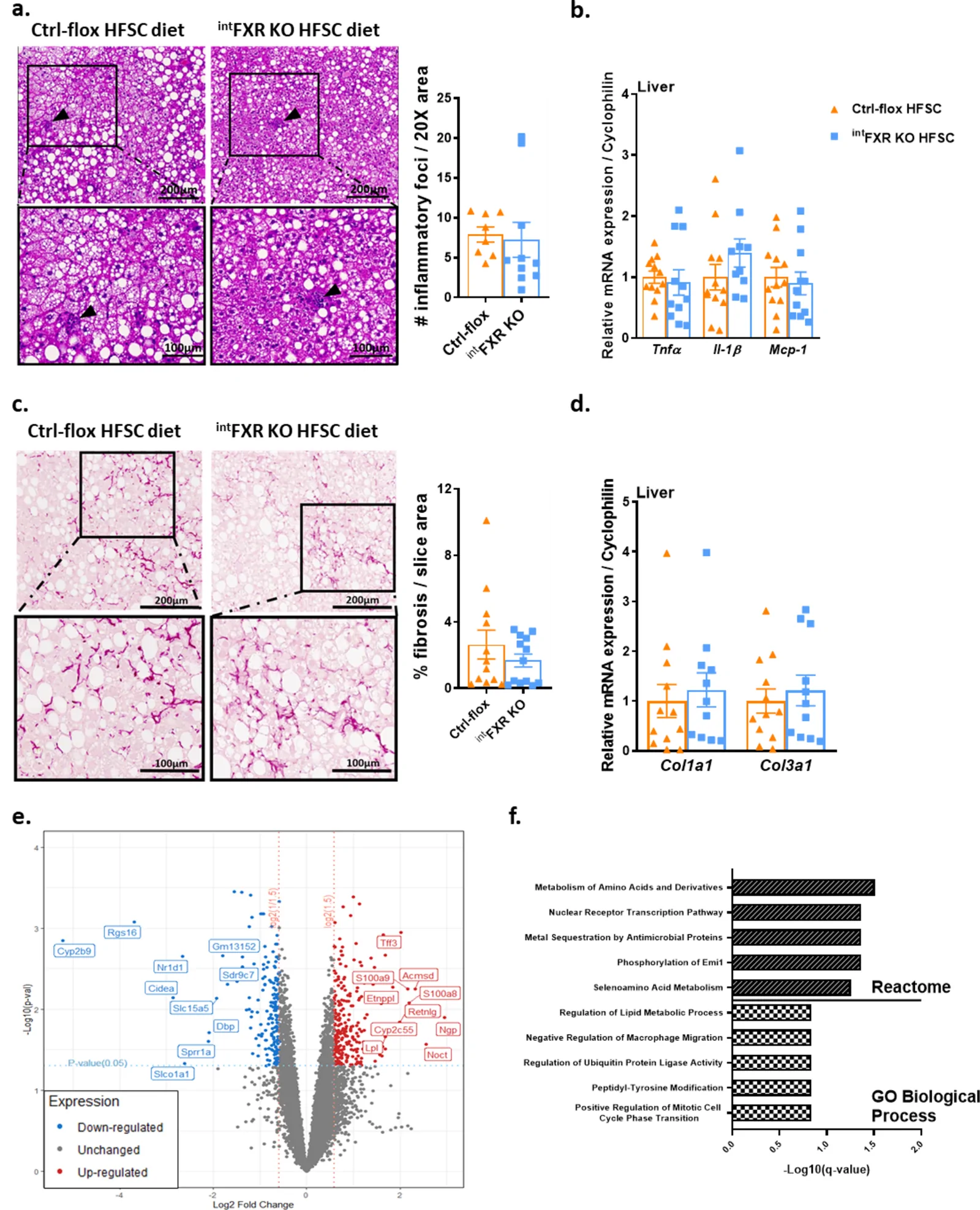
Effect of intestinal FXR deficiency on liver inflammation and fibrosis after 24 weeks of HFSC diet. **(a)** Representative hematoxylin and eosin (H&E)-stained liver sections and quantification of inflammatory foci in Ctrl-flox and ^int^FXR KO mice after 24 weeks of HFSC diet (*n* = 8–10/genotype). **(b)** qPCR quantification of selected inflammatory gene expression in whole liver tissue from Ctrl-flox and ^int^FXR KO mice after 24 weeks of HFSC diet (*n* = 8–12/genotype). **(c)** Representative Sirius Red-stained liver sections and quantification of fibrotic area in Ctrl-flox and ^int^FXR KO mice after 24 weeks of HFSC diet (*n* = 12/genotype). **(d)** qPCR quantification of selected fibrogenic gene expression in whole liver tissue from Ctrl-flox and ^int^FXR KO mice after 24 weeks of HFSC diet (*n* = 8–12/genotype). **(e)** Volcano plot showing differentially expressed genes in whole liver tissue from Ctrl-flox and ^int^FXR KO mice after 24 weeks of HFSC diet (*n* = 5/genotype). representing differentially expressed genes in total liver tissue of control (Ctrl-flox) and ^int^FXR KO mice after 24 weeks of HFSC diet (*n* = 5/genotype). **(f)** Pathway enrichment analysis of differentially expressed hepatic genes identified in Ctrl-flox and ^int^FXR KO mice after 24 weeks of HFSC diet (*n* = 5/genotype). For **(a-d)**, data are presented as mean +/- SEM and statistical significance was assessed using Mann–Whitney tests. *p < 0.05, ** *p* < 0.01. For **(e)**, genes were considered differentially expressed using a fold-change threshold of +/-1.5 and *p* < 0.05. (Abbreviation: Col1a1/Col3a1, alpha-1 type I (or III) collagen; H&E, Hematoxylin and eosin; HFSC diet, High-fat diet enriched with sucrose and cholesterol; Il-1β, Interleukin-1 β; Mcp-1, Monocyte chemoattractant protein 1;TNFα, Tumor necrosis factor α.)

As ^int^FXR KO mice are protected against weight gain and hepatic steatosis, both major drivers of liver inflammation and fibrosis, we next assessed the impact of intestinal FXR deficiency on progression toward MASH. Under chow diet conditions, only few inflammatory *foci* were detected in livers of both Ctrl-flox and ^int^FXR KO mice (Fig. S3e). HFSC diet feeding markedly increased inflammatory *foci* in both genotypes, with no significant difference detected by whole-slide quantification analysis (Fig. 5a). Consistently, hepatic expression of inflammatory genes, including *Tnfα*, *Il-1β* and *Mcp-1*, remained overall similar between genotypes (Fig. 5b). Liver fibrosis was absent in both genotypes under chow diet conditions (data not shown) and developed to a comparable extent following HFSC diet feeding, as assessed by Sirius red staining and expression of fibrogenic genes such as *Col1a1* and *Col3a1* (Fig. 5c&d). Blinded histopathological NAS assessment confirmed the protection against steatosis in ^int^FXR KO mice (Fig. S3f, *p* < 0.0074) with a moderate reduction in inflammatory *foci *compared with Ctrl-flox mice (*p* < 0.02). However, despite protection against steatosis and a moderate reduction in inflammatory *foci* by NAS scoring, fibrotic alterations remained present in ^int^FXR KO mice under HFSC feeding conditions.

Liver transcriptomics analysis from HFSC diet-fed ^int^FXR KO and Ctrl-flox mice identified 428 differentially expressed genes (Log2 fold change 0.585, *p* < 0.05) (Fig. 5e). In line with the lower hepatic steatosis in ^int^FXR KO mice, the lipid metabolism pathway was the most affected GO biological process pathway (Fig. 5f). Inflammatory pathways were also impacted in ^int^FXR KO mouse livers, as demonstrated by the negative regulation of the macrophage migration pathway (Fig. 5f). Thus, although there is no histological difference in macroscopical liver immune *foci*, there are likely subtle differences in cell composition and/or activity between ^int^FXR KO and Ctrl-flox mice.

### Impact of intestinal epithelium-specific FXR deficiency on hepatic immune cells in HFSC diet fed mice

**Fig. 6:**
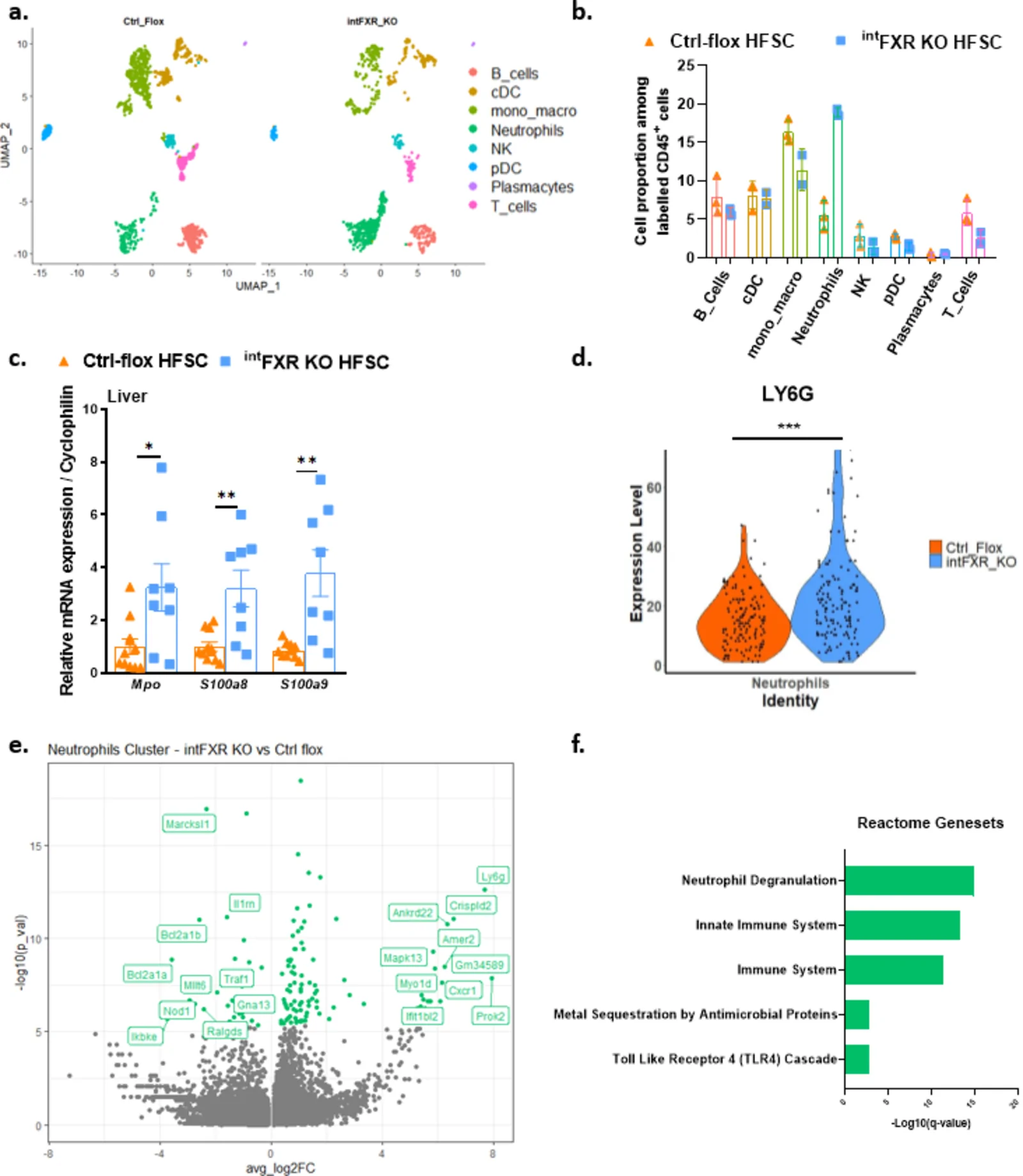
Effect of intestinal FXR deficiency on hepatic immune cell population and neutrophil-associated gene signatures assessed by scRNA-seq analysis. **(a)** UMAP representation of CD45^+^ cell subclusters identified by ScRNA-seq analysis in livers of ^int^FXR KO and Ctrl-flox mice after 24 weeks of HFSC diet. Cell numbers were downsampled to the lowest number of cells per genotype (*n* = 846 cells/genotype; *N* = 3 mice/genotype). **(b).** Relative proportions of each CD45^+^ cell cluster identified in **(a)** according to genotype. **(c)** qPCR quantification of selected neutrophil-associated gene expression in whole liver tissue from Ctrl-flox and ^int^FXR KO mice after 24 weeks of HFSC diet (*n* = 8–11/genotype). **(d)** CITE-seq quantification of LY6G protein expression in hepatic neutrophils isolated from Ctrl-flox and ^int^FXR KO mice after 24 weeks of HFSC diet. Neutrophil numbers were downsampled to the lowest number of cells per genotype (*n* = 3 mice/genotype). **(e)** Volcano plot showing differentially expressed genes in the neutrophil cluster between Ctrl-flox and ^int^FXR KO mice after 24 weeks of HFSC diet (Adj-p value < 0.05). **(f)** Top five enriched pathways identified from differentially expressed genes in the neutrophil cluster (pathway p-value < 0.05). For **(c)**, data are presented as mean ± SEM and statistical significance was assessed using Mann–Whitney tests. *p < 0.05, **p < 0.01. (Abbreviations: cDC, conventional dendritic cell, HFSC diet, High-fat diet enriched with sucrose and cholesterol, pDC, plasmacytoid dendritic cell; NK, natural killer; UMAP, Uniform Manifold Approximation and Projection).

To assess at higher granularity the impact of intestinal FXR deficiency on liver immune cells, we next studied the impact of the MASH diet-feeding on hepatic immune cells by scRNA-seq analysis of CD45^+^ cells sorted from livers of HFSC diet fed ^int^FXR KO and Ctrl-flox mice. Unbiased clustering analysis identified 8 leukocyte populations (Fig. 6a), annotated on the basis of their gene expression (Fig. S4a). The proportion of cells in the conventional dendritic cell (cDC) cluster, known to be altered upon MASH development^28^, was similar between HFSC diet fed ^int^FXR KO and Ctrl-flox mice (Fig. 6b). However, the cDC1:cDC2 ratio, which is decreased in MASH livers^24^, was higher in ^int^FXR KO livers (Fig. S4b&c). Moreover, the proportion of T lymphocytes (Fig. 6b and Figure S4d) was lower in ^int^FXR KO mouse livers (Fig. S4e), whereas an increased CD4:CD8 ratio was observed, mainly due to a reduced number of CD8^+^ cells. These differences between ^int^FXR KO and Ctrl-flox mice may be indicative of a lower immune adaptive response after HFSC diet feeding in livers of ^int^FXR KO mice.

In contrast, the neutrophil cluster was proportionally increased in livers from ^int^FXR KO mice compared to Ctrl-flox mice (Fig. 6b). In line, whole liver transcript levels of *Mpo*, *S100a8*, *S100a9* (Log_2_ FC = 0.57, *p* = 0.0328 for *Mpo*; Log_2_ FC = 0.97, *p* = 0.0091 for *S100a8* and Log_2_ FC = 1.07, *p* = 0.0021 for *S100a9*) were increased in these mice (Fig. 6c). CITE-seq analysis showed a higher expression of the neutrophil marker protein LY6G, indicating an increase of neutrophils in ^int^FXR KO compared to Ctrl-flox mice livers (Fig. 6d, *p* < 0.001). Differential gene expression analysis of neutrophil cluster cells identified 135 genes differentially expressed between neutrophils from ^int^FXR KO and Ctrl-flox mice, with 103 up-regulated genes in the ^int^FXR KO genotype (Fig. 6e). Pathway analysis of the differentially expressed genes indicated altered neutrophil functions, with an increased expression of neutrophil degranulation-related genes, such as *S100a8*, *S100a9*, *Fpr1* and *Lcn2* (*adj-p values = 3.13 × 10*^*− 11*^; *3.26 × 10*^*− 15*^; *7.57 × 10*^*− 5*^ *and 0.03* respectively) in ^int^FXR KO mice (Fig. 6f), indicative of the presence of activated neutrophils in livers of HFSC diet-fed ^int^FXR KO mice. Neutrophil sub-clustering analysis identified 3 major neutrophil subclusters (Fig. S4f&g), mainly consisting of the *Atcg1*^*+*^ and *Mmp8*^*+*^ neutrophil clusters, which have been associated with liver tumorigenesis^29^. The increased proportion of neutrophils in ^int^FXR KO mice was not related to particular neutrophil subclusters. Altogether, these results indicate that intestinal FXR deficiency results in a distinct hepatic immune signature, indicative of a switch from an adaptive to innate immune response.

## Discussion

The present study demonstrates that intestinal FXR deficiency dissociates protection against obesity and hepatic steatosis from protection against hepatic inflammation and fibrosis during HFSC-induced MASH progression. These findings highlight the complexity of gut-liver FXR signaling and suggest that metabolic improvement alone may be insufficient to prevent inflammatory and fibrotic liver remodeling. More generally, our data emphasize that intestinal FXR modulation may differentially affect metabolic and immune components of MASH pathogenesis.

Hepatic inflammation is a major driver of progression from MASL to MASH and is associated with deep remodeling of the liver immune cell profiles due to the recruitment of CCR2^+^ monocyte-derived macrophages^30,31^ and CD8^+^ T cells^32^ or deregulation of conventional dendritic cell profiles^28^. In the present study, scRNA-seq analysis identified that intestinal FXR deficiency did not normalize the pro-inflammatory hepatic immune profile induced by the HFSC diet, despite protection against obesity and hepatic steatosis. Instead, ^int^FXR KO mice displayed increased hepatic neutrophil proportions associated with altered distributions of monocyte and macrophage populations, suggesting persistent immune activation with a strong antimicrobial response independently of lipid accumulation.

Liver neutrophil recruitment is a key contributor to MASH development and fibrotic remodeling^33^. Recent studies have highlighted the contribution of innate inflammatory pathways, including oxidative stress- and inflammasome-associated mechanisms, to hepatic injury and fibrosis progression, further supporting the importance of immune remodeling during chronic liver disease^34^. Previous studies demonstrated that overexpression of the chemokine CXCL1 in obese HFD-fed mice enhances hepatic neutrophil infiltration, inflammation, and fibrosis, promoting MASL to MASH transition^35^. Beyond MASH, neutrophils also contribute to other liver pathologies, including acute-on-chronic liver failure^36^ and hepatocarcinoma^37^. In this context, the increased expression of neutrophil-associated and degranulation-related genes observed in ^int^FXR KO mice may reflect sustained innate immune activation despite reduced steatosis. Although the mechanisms linking intestinal FXR deficiency to hepatic neutrophil enrichment remain unresolved, these observations raise the possibility that gut-derived inflammatory signals or microbiota-associated factors may contribute to persistent hepatic immune remodeling in this model.

Intestinal FXR deficiency was associated with multiple alterations affecting intestinal homeostasis under HFSC diet condition. In particular, a decreased expression of several barrier function-related genes was observed both in intestinal tissues and intestinal organoids derived from ^int^FXR KO mice. Whereas chow diet-fed whole-body FXR KO mice display bacterial infiltration, intestinal edema and a pronounced intestinal permeability^25^, we did not observe any tissue structure abnormalities between both genotypes fed a MASH-like diet. These data, supported by the lack of difference in the less sensitive FITC-dextran assay^38^, suggest that intestinal FXR deficiency may result in subtle epithelial remodeling rather than severe barrier disruption. Additional approaches including circulating endotoxin measurements^39,40^ would be required to further investigate whether increased microbial-derived low-grade inflammatory signals contribute to the persistent hepatic immune activation observed in ^int^FXR KO when fed the HFSC diet.

Transcriptomic analysis further revealed modifications in BA metabolism related pathways, consistent with the established roles of intestinal FXR in regulating the enterohepatic BA cycle^26^. Although only modest differences in BA composition were observed between both genotypes, increased levels of 6α-hydroxylated BA, including HCA and HDCA, may reflect altered microbiota-dependent BA metabolism. Consistent with this hypothesis, ^int^FXR KO mice displayed an increased abundance of bacterial taxa belonging to the *Lachnospiraceae* family, which include species capable of BA epimerization necessary for HCA production^41,42^. Previous studies reported beneficial metabolic effects associated with HCA-producing bacteria in western diet fed mice^43^, suggesting that alterations observed in the liver are not completely dependent on the improved metabolic phenotype driven by the BA-microbiota interaction. In addition, the relationship observed here between microbiota composition, BA remodeling, and the metabolic phenotype remains strictly correlative and a possible causal relation requires additional dedicated studies.

In parallel, intestinal FXR deficiency was associated with altered intestinal immune cell populations, including increased intraepithelial CD8^+^ T cell, particularly the CD8^+^TCRγδ^+^ T population, which is involved in protection of the intestinal epithelium against bacterial infections^44^. Although this population has not been directly linked to MASLD pathology, it has been shown to exacerbate colitis in mouse models^45,46^. Moreover, B cells, which contribute to intestinal barrier protection and immune tolerance through IgA production^27,47^, are reduced, along with decreased plasma IgA levels. These observations suggest altered intestinal immune homeostasis in ^int^FXR KO mice fed the HFSC diet. Nevertheless, whether these immune alterations arise directly from intestinal FXR deficiency, indirectly from microbiota remodeling, or from other diet-associated factors remains unresolved.

The present study was performed using a long-term HFSC diet-induced MASH model, which recapitulates key metabolic and hepatic features associated with human disease^24^. While additional models, such as chemical treatment or choline- or methionine-deficient MASH-inducing diets,^48,49^ or genetic models may help to further clarify the mechanisms underlying the observed phenotype, our approach allowed us to investigate the consequences of intestinal FXR deficiency in the context of integrated metabolic dysfunction and chronic liver injury.

Our data are consistent with previous studies showing protective effects of intestinal FXR deficiency against obesity and hepatic steatosis in a HFD model^16,17,19^. Similar metabolic improvements have also been described in whole-body FXR KO mice, whereas hepatic FXR deficiency does not appear to confer protection against obesity nor metabolic disorders^15^. Together, the observations support that intestinal FXR signaling may differentially regulate metabolic and hepatic inflammatory pathways during MASH progression. Several mechanisms may contribute to these tissue-specific effects. Previous studies suggested that intestinal FXR inhibition may enhance GLP-1 production^50^, thereby improving hepatic steatosis, whereas reduced intestinal FGF15 signaling alters hepatic BA regulation and liver repair^4^. Although these pathways were not directly investigated in the present study, they may contribute to the observed dissociation between liver steatosis protection with persistence of hepatic inflammation and fibrosis.

Interestingly, clinical studies targeting FXR-related pathways have also reported heterogeneous hepatic outcomes. FGF19-based therapies such as aldafermin improved hepatic steatosis but showed limited effects on fibrosis^14^. By contrast, FXR agonists such as OCA reduce fibrosis without consistently improving overall MASH resolution in patients^6^. However, altered bile acid homeostasis, dysregulated FXR signaling, and gut microbiota remodeling have also been reported in patients with MASLD/MASH and have been associated with disease severity and fibrosis progression^51–54^. In particular, changes in bile acid composition and gut microbial signatures have been linked to disease severity and metabolic dysfunction in MASH patients^53^. Regretfully, these clinical observations, although heterogeneous across studies, highlighted the contribution of intestinal FXR signaling to human MASH, even if the details remain incompletely understood. Therefore, further translational studies will be required to determine the extent to which the mechanisms identified in the present murine model are relevant in patients.

While the present study provides insight into the consequences of intestinal FXR deficiency during diet-induced MASH progression, several limitations should be acknowledged. First, the mechanistic links between intestinal FXR deficiency, microbiota remodeling, altered intestinal immunity and hepatic neutrophil enrichment remain associative. Dedicated causal approaches, including microbiota transfer or targeted gut-liver axis interventions, will be required to determine the precise contribution of each of these pathways to the observed phenotype. Second, although transcriptional alterations affecting intestinal barrier-associated genes were identified, no overt increase in intestinal permeability was detected using the FITC-dextran assay. Additional approaches with higher sensitivity, including circulating endotoxin measurements, may help to further clarify whether subtle barrier alteration contribute to persistent hepatic inflammation in this model. Third, the present work was conducted using a single long-term HFSC diet MASH model and was not specifically designed to comprehensively assess systemic metabolic parameters such as insulin resistance, adipose tissue remodeling, or pair-feeding effects. Further studies integrating these complementary analyses across multiple experimental models would help to refine the interpretation of the metabolic and inflammatory dissociation observed in ^int^FXR KO mice. Finally, the scRNA-seq analyses were performed on a limited number of animals and should therefore be interpreted primarily as an exploratory characterization of hepatic immune remodeling associated with intestinal FXR deficiency.

To conclude, our study identifies a distinct hepatic phenotype associated with intestinal FXR deficiency under MASH-inducing conditions. Despite protection against obesity and hepatic steatosis, ^int^FXR KO mice still develop hepatic inflammation and fibrosis, supporting the concept that steatosis and inflammatory/fibrotic progression may dissociate during MASH development. Although the precise mechanisms underlying this phenotype remain unresolved, several hypotheses may be considered for future investigation. In the context of HFSC feeding, altered intestinal barrier as described in FXR-deficient models^25^, together with diet-associated microbiota remodeling, may increase exposure to microbial-derived inflammatory signals, including endotoxin-related PAMPs^39^. These signals are known to contribute to hepatic neutrophil recruitment and activation^55^, and could therefore participate in the persistence of hepatic inflammatory and fibrotic remolding despite reduced steatosis.

While these mechanisms remain hypothetical and will require dedicated mechanistic investigation, our findings highlight the complexity of gut-liver FXR signaling and suggest that intestinal FXR modulation alone may not be sufficient to fully prevent MASH progression.

## Methods

### Mouse model

Intestine-specific FXR KO (^int^FXR KO) and littermate control (FXR^*fl/fl*^ or Ctrl-flox) mice on a C57BL/6J genetic background were obtained using the Cre-lox strategy by crossing FXR-floxed mice from F. Gonzalez^16^ with Villin-Cre mice^56^. Mice were maintained in a specific pathogen-free environment (12:12 h light/dark cycle, 21–24 °C) with *ad libitum* access to water and food. Eight-week-old male mice were randomized by body weight prior to the start of either a control diet (CD, standard rodent chow, 5% kcal fat, Safe A04; SAFE diets, Augy, France) or a MASH-inducing diet (HFSC, 45% kcal fat, 40% kcal carbohydrate, 15% kcal protein with 1% (by weight) cholesterol; SAFE diets, Augy, France) for 24 weeks. Body weight was recorded every week on Tuesday at 6.00pm. Blood sampling for biochemical analysis was carried out after a 4 h fast. Mice were euthanized by cervical dislocation without anesthesia at the fed state. The number of mice in each group is stated in the legend of the figures. All animal experiments were approved by the Ethics Committee for Animal Experimentation of the Hauts-de-France Region (Approval N°: APAFIS#3939-2015212152254444671 v8). All animal studies were performed in accordance with the institutional guidelines for the care and use of laboratory animals, national regulations and the ARRIVE guidelines (https://arriveguidelines.org/).

### Ileum-derived mouse organoids

Eight-week-old ^int^FXR KO and littermate control mice were euthanized by cervical dislocation without anesthesia. Ileum segment was harvested and flushed with cold PBS. Crypts were separated by pipetting, digested using the manufacturer’s solution Gentle Cell Dissociation Reagent (STEMCELL Technologies #100–0485), and incubated at room temperature for 15 min on a rocking platform. Tissue fragments were resuspended in PBS containing BSA 0.1% and mechanically dissociated. Crypts were collected, passed through a 70 μm filter, and centrifuged at 290 × g for 5 min at 4 °C. The crypt pellet was washed once with advanced DMEM/F-12 (Gibco #12634010) containing 15 mmol/L HEPES. Approximately 500 crypts were embedded in Matrigel Matrix (Corning, #356231) and seeded in 48-well plates. Organoids were cultured in IntestiCult Organoid Growth Medium (Mouse) (STEMCELL Technologies #06005), supplemented with 1% gentamicin (Gibco #15710049) and 10 µM Y-27,632 (Merck #Y0503-1MG).

After a few passages, the organoids were seeded in matrigel 6 days prior to the experiments. Lipoglucotoxic treatments were previously described by Boeckmans et al.^57^.

### Histology

Mouse liver sample sections were fixed in 4% paraformaldehyde during 20 h (P6148 – Sigma Aldrich), embedded in paraffin, cut at 3 μm and stained with hematoxylin-eosin or Sirius red as previously described^24^. Images were obtained by slide scanner (Axioscan Z1 - Zeiss). Steatosis and fibrosis quantification were performed with Image J software (Version 1.53) using Javascript langage (BioImaging Center Lille, BICeL - UMS 2014 – Lille University). The inflammatory foci were counted by two blinded experimenters in 10 areas of 20X distributed on the hematoxylin-eosin-stained sections (Zeiss Efficient Navigation – Zeiss). Simple foci close to vessels were excluded as considered blood circulating inflammatory cells.

### RNA extraction

Total RNAs were isolated from mouse liver and intestine using TRIzol^®^ reagent (Invitrogen #15596018). Total RNA from ileum-derived mouse organoids was extracted using the RNesay Plus Micro Kit (Quiagen, #74034). Purified RNA was then used for reverse transcription and real-time PCR. For samples used for microarray, quantification and RNA integrity number (RIN) were tested using the Agilent 2100 Bioanalyzer System. Only RNA samples with RIN ≥ 6.8 were used.

### Reverse transcription and real-time PCR

Total RNA (1,000 ng) were treated with DNAse I and used to generate cDNA with High-capacity cDNA reverse transcription kit (Applied Biosystems #4368813). Gene expression was measured by real-time PCR using Master mix PowerTrack SYBR green (Applied Biosystems # A46109). Results were normalized to the normalization factor calculated as average expression of housekeeping gene cyclophilin, and the ΔΔCt method was used for analyses. Primer sequences are provided in Table S2.

### Microarray analysis

Transcriptomics analysis was performed with Clariom™ S Assay, mouse^24,58^ All liquid handling procedures were performed on a GeneChip Fluidic Station 450. GeneChips were scanned with a GeneChip Scanner 3000-7G (Affymetrix) using Command Console v4.1.2. Quality controls were performed using the Affymetrix expression console. Transcriptomics data were extracted by informatic format (CEL). Microarray analyses were performed with the GIANT tools suite^59^ on a local instance of Galaxy^60^. Data were normalized with Affymetrix Power Tool in GIANT (APT, ThermoFisher Scientific, 2018) using “GC, RMA and Scale intensity” as parameters and pathway studies were made by EnrichR, focusing on the Reactome and GO Biological Process database (https://maayanlab.cloud/Enrichr/). Significant pathways from the top 10 are sorted by -log10(q-Value).

### Western blot analysis

Proteins were extracted from mouse intestine and liver using B buffer (PBS 1X, SDS 0.1%, NaDoc 0.5%, Igepal 1%, Complete 1X (Roche)) or lysis buffer (Tris 50 mM at pH 8, NaCl 137 mM, Glycerol 10%, NP40 1%, phosphatase inhibitors (NaF 1 M, β-glycerolphosphate 1 M, Na_3_(VO)_4_ 0.5 M), Complete 1X (Roche), H_2_0) respectively. After sonication, proteins were dosed by the BCA kit (Thermo Scientific). Forty µg proteins in Laemmeli buffer (Tris-HCl 62.5 mM, SDS 2%, glycerol 10%, bromophenol bleue) were denatured at 95 °C for 5 min. Blots were generated following the protocol previously described^61^ and incubated with FXR (PP-9033 A, R&D) or β-actin (SC-1616, Santa Cruz) antibodies diluted at 1:500. After 1-hour incubation with horseradish peroxidase-conjugated secondary antibodies diluted at 1:3,000 (Sigma), revelation was performed using the enhanced chemiluminescence FEMTO Plus reagents (ECL FEMTO, ThermoFisher) by autoradiography (Camera Gbox, SynGene) and band intensities were measured (GeneTools software, SynGene).

### Biochemical assays

Plasma transaminases AST and ALT were quantified by enzymatic methods using ready to use reagents (ThermoFisher #MG981771 and #MG981769) adapted to automate (Konelab 20 – Thermo Scientific) according to manufacturer’s instructions. Hepatic cholesterol and triglycerides were quantified from 50 mg of liver. Samples were grinded in set buffer (Sucrose 250 mM, Tris-base 10 mM, EDTA 2 mM) and underwent 2 freeze-thaw cycles each followed by syringe pressing. The assays were performed by photometry (Konelab 20 – Thermo Scientific).

Cholesterol and triglycerides were measured by enzymatic methods using ready to use reagents (DiaSys, Cholesterol* FS, CQN: ET and DiaSys, Triglycerides FS*, #1 5710 99 10 923) adapted to automaton (Konelab 20 – Thermo Scientific) according to manufacturer’s instructions.

Circulating Immunoglobulins A (IgA) in serum from mouse caudal vein were quantified by ELISA kit (SBA Clonotyping System-C57BL/6-HRP – 5300-05B – SouthernBiotech). Standard range was made by successive purified IgA dilutions (Mouse Immunoglobulin Panel – 5300-01 - SouthernBiotech) and the revelation substrate was TMB (3,3,5,5’-Tetramethylbenzidine). Quantification was carried out using spectrophotometry (Infinite M200Pro – Tecan) according to manufacturer’s instructions.

### Isolation of immune cells from intestinal tissues and flow cytometry

Immune cells were isolated from epithelium and *lamina propria* of mouse small intestine^62^. Whole small intestines were washed in cold PBS 1X and cut in 1 cm² pieces. The intra-epithelial cells (IEC) were obtained in supernatant after sample incubation in dithioerythritol (DTE) solution (Hanks’ Balanced Salt Solution (HBSS) without Ca^2+^ and Mg^2+^ (Gibco #14170-088); fetal calf serum 10% (Dutscher #500105H1); HEPES 15 mM (H4034 – Sigma); DTE 1 mM (Alfa Aesar # A10138)) at 37 °C, 20 min at 200 rpm followed by two filtrations at 70 μm. Intestinal pieces were further incubated in EDTA solution (HBSS without Ca^2+^ and Mg^2+^ (Gibco #14170-088); fetal calf serum 5% (Dutscher #500105H1); HEPES 15 mM (Sigma # H4034); EDTA 5 mM (Sigma)) at 37 °C, 30 min at 200 rpm followed by collagenase IV treatment (HBSS without Ca^2+^ and Mg^2+^ (Gibco #14170-088); fetal calf serum 10% (Dutscher #500105H1); HEPES 15 mM (Sigma # H4034); Collagenase IV 2 mg/mL (Gibco #17104-019)) 90 min at 37 °C at 110 rpm and a filtration at 70 μm to isolate cells from *lamina propria*. IEC and immune cells from *lamina propria* were separated from other cells by 40–80% Percoll (GE Healthcare # GE17-0891-01) gradient, washed in PBS 1X, and resuspended in FACS buffer (PBS 1X with 0.5% of BSA). Cell viability was checked with the Automated Cell Counter (TC20™ – Bio-Rad) after trypan blue labelling (Gibco #15250–061). Cells were incubated with Zombie UV (BUV 496, Biolengend #423107/8) at 1:100 (final dilution) to discriminate live and dead cells, incubated with Fc-block (CD16/30–553142 – BD Biosciences) at 1:125 and labelled with conjugated antibodies (Table S3). Flow cytometry analysis was performed on BD LSRFortessa X-20 (Becton Dickinson). Results were acquired with the Diva software (Becton Dickinson) and analyzed using FlowJo software (Tree Star).

### BA quantification

BA quantification in caecal content and liver was performed as previously described^63,64^. Briefly, caecal content or liver was lyophilized and homogenized in NaOH. Then BA were extracted by solid-phase extraction (SPE, Oasis HLB 3 cc 60 mg: WATERS #WAT094226) using methanol for elution as previously described ^64^.LC–MS/MS analysis was performed on a UFLC-XR HPLC device (Shimadzu) coupled to a QTRAP^®^ 5500 LC/MS/MS system hybrid triple quadrupole/linear ion trap mass spectrometer (AB Sciex, Foster City, CA, USA) equipped with a Turbo VTM ion source. The method allowed the quantification of 21 different BA within the range of 1.5-2,000 nmol/L (cholic acid (CA), glycocholic acid (GCA), taurocholic acid (TCA), chenodeoxycholic acid (CDCA), glycochenodeoxycholic acid (GCDCA), taurochenodeoxycholic acid (TCDCA), deoxycholic acid (DCA), glycodeoxycholic acid (GDCA), taurochenodeoxycholic acid (TCDCA), lithocholic acid (LCA), glycolithocholic acid (GLCA), taurolithocholic acid (TLCA), ursodeoxycholic acid (UDCA), glycoursodeoxycholic acid (GUDCA), tauroursodeoxycholic acid, (TUDCA), α-muricholic acid (αMCA), tauro-α-muricholic acid (TαMCA), β-muricholic acid (βMCA), tauro-β-muricholic acid (TβMCA), ω-muricholic acid (ωMCA), tauro-ω-muricholic acid (TωMCA), hyocholic acid (HCA), glycohyocholic acid (GHCA), taurohyocholic acid (THCA), hyodesoxycholic acid (HDCA), taurohyodesoxycholic acid (THDCA), and glycohyodesoxycholic acid (GHDCA)), using an internal standard containing deutered forms (CA-D4, GCDCA-D4, CDCA-D4, TCA-D4, GCA-D4, Tyr-CA-D4 and Leu-CA-D4). Instrument control, data acquisition and processing were performed using the associated Analyst 1.5.1 software. The separation was carried out on a Symmetry C18 Luna column (250 mm×2.1 mm, particle size 5 μm) (Phenomenex). The injection volume was 5 µL. Elution was performed at a flow rate of 500 µL/min with water containing 20 mM ammonium acetate, pH = 8 as eluent A and acetonitrile as eluent B, employing a 5 min plateau with 28% B and a linear gradient from 28% B to 90% B in 15 min, followed by a 2 min plateau with 90% B. Then, column re-equilibration was performed for 4 min. The injection duty cycle was 26 min, considering the column equilibration time.

### Gut microbiota analysis

DNA was extracted from caecal content following the protocol Q^65^. This protocol uses the QIAamp DNA Stool Mini Kit (Qiagen, Germany) and includes a bead-beating step. The DNA was analyzed using 16 S rRNA gene sequencing. Sequencing procedures, as well as bioinformatics and biostatistics analyses, are detailed in the Supporting Information. Raw sequences can be found in the SRA database (projects ID: PRJNA1091900 https://dataview.ncbi.nlm.nih.gov/object/PRJNA1091900?reviewer=fpf5msfpbv0g2s36cvj2t5dhab).

### Single cell RNA-seq and CITE-seq on liver immune cells

Immediately after sacrifice, liver was flushed by injection of 5 mL of cold PBS 1X in the portal vein, dissected and weight recorded for normalization. Samples were minced in digestion media (collagenase D at 1 mg/mL (Sigma #11088882001) in RPMI) and incubated 20 min at 37°C with shaking. Cell suspensions were gently passed by syringe through a 19G needle five times filtered (70 µm) and washed with FACS buffer. After centrifugation, the supernatants were gently decanted and the pellets were resuspended in cold red blood cell lysis buffer (RBC). After washing with FACS buffer and centrifugation (5 min, 13,200 rpm, 4°C), cells were labelled with Zombi Green (Biolengend #423107/8) and CD45.2 antibody (BV510). Live immune cells were isolated by sorting (BD Influx Cell sorter – BD Biosciences). Samples were prepared for single cell RNA-Sequencing with the Chromium Next GEM Single cell 3’ Reagent Kit (Dual Index) (10x genomics). Cells from all mice were labelled using tagged antibodies for CITE-seq protein expression (Supplementary table S4) and Cell Multiplexing Oligos then treated in multiplexing following 10X genomics protocols to construct library upon sequencing. Final sequencing to an average depth of 100k reds/cell was performed by BGI (Hong Kong).

### Preprocessing scRNA-seq

After sequencing quality control, CellRanger v6.0.2 (10X Genomics, USA) was used to align reads (excluding intronic reads) to the mouse reference genome mm10-2020-A obtained from 10X Genomics and generate the UMI counts matrix. The Seurat v.4.0.3 R package^66^ was used for further analysis of the raw counts. For quality control, only cells expressing between 200 and 5000 genes were included as well as cells with less than 8% mitochondrial genes. Initial clustering normalized by logNormalize function (NormalizeData) was performed on the PCA-reduced dataset (ndims = 20) using Seurat FindNeighbors and FindClusters commands at a resolution of 0.3. The specific genes of each cluster were identified with the FindAllMarkers function with parameters min.pct = 0.01, test.use = ‘wilcox’. Clusters were manually annotated according to cell type-specific genes, combined with marker genes from literature reports.

### Statistical analysis

Data are presented as means ± SEM. Outliers were removed based on Grubbs’ test. Microarray statistical analyses were performed with Galaxy software and Giant extension. Hepatic dysregulated genes were defined using Benjamini-Hochberg corrected p-value cut-off set at 0.15, allowing to define genes numerous enough for robust downstream analysis with EnrichR software (https://maayanlab.cloud/Enrichr/), and a minimal fold change of 1.2. Data statistical analyses were performed with Prism 9 (GraphPad Software, Inc.), R (version 4.1) or MetaboAnalyst (Version 6.0) for spearman’s correlations. When comparing 2 groups, unpaired-T tests or Mann-Whitney were used after checking for normality. When comparing more than 2 groups, one-way or two-way ANOVA followed by post-hoc Sidak correction or Kurskal-Wallis followed by Dunn’s multiple comparisons tests were used. The appropriate statistical test used are specified in the corresponding figure legend. A p-value < 0.05 was considered significant.

## Supplementary Information

Below is the link to the electronic supplementary material.

Supplementary Material 1

## Data Availability

The intestinal and liver Affymetrix microarray data, as well as the liver immune single-cell RNA-seq data generated in this study, have been deposited in the NCBI Gene Expression Omnibus (GEO) under accession numbers GSE318026 and GSE318468, respectively.

## Abbreviations

ALT: Alanine aminotransferase
AST: Aspartate transaminase
ASV: Amplicon Sequencing Variants
BA: Bile acids
cDC: Conventional dendritic cells
Ctrl-flox: Fxr^fl/fl^ mice
DC: Dendritic cells
DCA: Deoxycholic acid
DSS: Dextran Sulfate Sodium
FGF 15/19: Fibroblaste Growth Factor 15/19
FXR: Farnesoid X Receptor
GLP1: Glucagon-Like Peptide 1
HCA: Hyocholic acid
HFD: High-fat diet
HFSC diet: High-fat diet enriched with sucrose and cholesterol
IgA: Immunoglobulin A
FXR KO: Intestinal FXR-deficient
MASH: Metabolic dysfunction-associated steatohepatitis
MASLD: Metabolic dysfunction-associated steatotic liver disease
OCA: Obeticholic acid
PCA: Principal component analysis
TG: Triglyceride
T: T lymphocyte
