# Supplementary material for: Intestinal FXR deficiency uncouples steatosis protection from liver inflammation and fibrosis in MASH-diet fed mice
Source: Sci Rep. 2026 Jul 10;16:31762. doi: 10.1038/s41598-026-60976-y (PMC13654801; doi:10.1038/s41598-026-60976-y)
Supplement: Supplementary file 1 [file 41598_2026_60976_MOESM1_ESM.docx]

**Supplementary data**

**Intestinal FXR deficiency uncouples steatosis protection from liver inflammation and fibrosis in MASH-diet fed mice**

Margaux Nawrot^1,*^, Simon Peschard^1,*^, Morgane M Thibaut^2^, Kévin Ory^1^, Emmanuelle Vallez^1^, Emilie Dorchies^1^, Véronique Touche^1^, Julie Dubois-Chevalier^1^, Céline Gheeraert^1^, Sandrine Caron^1^, Kadiombo Bantubungi^1^, Philippe Lefebvre^1^, Anne Tailleux^1^, Joel T Haas^1^, Laure B Bindels^2,3^, Isabelle Leclercq^4^, David Dombrowicz^1^, Sophie Lestavel^1,#^, Bart Staels^1,#^

**Supplementary tables**

*Supplementary Table S1: Genes differentially expressed between the ileum of ^int^FXR KO and Ctrl-Flox mice fed the HFSC diet for 24 weeks.* Fold-change +/- 1.2; FDR P-value < 0.15.

*Supplementary Table S2: Primers used for qPCR***.** Primers with hybridation temperature close to 60°C were *In Silico* chosen using the NCBI-BLAST software (<https://blast.ncbi.nlm.nih.gov/Blast.cgi> ).

| **Gene** | **5’→3’ forward** | **5’→3’ reverse** |
| --- | --- | --- |
| ***Cldn2*** | TGAACACGGACCACTGAAAG | TTAGCAGGAAGCTGGGTCAG |
| ***Cyclophilin*** | GCATACGGGTCCTGGCATCTTGTCC | ATGGTGATCTTCTTGCTGGTCTTGC |
| ***Cyp7a1*** | ATGCTTCTGTGTCCAAATGCC | CTGTCATACCACAAAGTCTTATGTCA |
| ***Col1a1*** | AGCACGTCTGGTTTGGAGAG | GCAGTGATAGGTGATGTTCTGG |
| ***Col3a1*** | TGGCAATGTAAAGAAGTCTC | CCCAGTGTGTTTAGTACAGC |
| ***Fgf15*** | GAGGACCAAAACGAACGAAATT | ACGTCCTTGATGGCAATCG |
| ***Nr1h4 (Fxr)*** | CCTGAGAACCCACAGCATTT | GTGTCCATCACTGCACATCC |
| ***Jam-a*** | AGCCGGGAGGAAACTGTTGT | GCAGGTCAATTTGATGGACTCGT |
| ***Il1β*** | AGGTGCTCATGTCCTCATCC | CAGGCAGGCAGTATCACTCA |
| ***Lyz1*** | GGTCTACAATCGTTGTGAGTTGG | AAACACACCCAGTCAGCCAG |
| ***Mcp-1*** | GCCAACTCTCACTGAAGCC | GCTGGTGAATGAGTAGCAGC |
| ***Mpo*** | ATCATCACATACCGGGACTACC | TTCGAGGGTCTACTGAGTCGTT |
| ***Muc2*** | CCTGAAGACTGTCGTGCTGT | GGGTAGGGTCACCTCCATC |
| ***Occludin*** | AAGAGCTTACAGGCAGAACT | CTGCAGATCCCTTAACTTG |
| ***Nr0b2 (Shp)*** | ACGATCCTCTTCAACCCAGA | AGGGTAGAGGCCATGAGGAG |
| ***S100a8*** | CCGTCTTCAAGACATCGTTTGA | GTAGAGGGCATGGTGATTTCCT |
| ***S100a9*** | AATGGTGGAAGCACAGTTGG | CTGGTTTGTGTCCAGGTCCTC |
| ***Tff3*** | TTTGACTCCAGTATCCCAAATGT | TGCAGAGCTCCCTTCCT |
| ***Tnfα*** | CCCCAAAGGGATGAGAAGTT | CACTTGGTGGTTTGCTACGA |
| ***Zo-1*** | CGCTAAGAGCACAGCA | AGGATTATCTCTTCCACCAG |

*Supplementary Table S3: Antibodies used for FACS analyses.*

(Abbreviations: AF700, Alexa Fluor 700; APC, Allophycocyanin; BUV, Brillant ultraviolet; BV, Brillant violet; CD, Cluster of differentiation; FITC, Fluorescein isothiocyanate; Ig, Immunoglobulin; MHC, major histocompatibility complex; PE, phycoerythrin; TCR, T-cell receptor; UV, ultraviolet)

| **Target / marker** | **Fluorochrome** | **Isotype** | **Dilution** | **Reference** |
| --- | --- | --- | --- | --- |
| **CD11b** | BUV 395 | Rat IgG2b. κ | 1/1000 | BD Biosciences 563553 |
| **CD45.2** | BUV 737 | Human IgG2a. κ | 1/200 | BD Biosciences 564880 |
| **Zombie -UV** | BUV 496 | - | 1/100 | Biolegend 423107/8 |
| **TCR αβ** | BV 510 | Hamster Ig. λ | 1/100 | Biolegend 109233 |
| **TCR γδ** | BV 421 | Hamster IgG | 1/100 | Biolegend 118120 |
| **CD4** | BV 605 | Rat IgG2a. κ | 1/200 | Biolegend BLE100548 |
| **MHC II - IA/IE** | BV 650 | Rat IgG2b. κ | 1/1500 | Biolegend BLE107641 |
| **CD19** | BV 711 | Rat IgG2a. κ | 1/300 | BD Biosciences 563157 |
| **F4/80** | BV 786 | Rat IgG2a. κ | 1/200 | Biolegend BLE123141 |
| **CD11c** | FITC | Hamster Ig. λ | 1/100 | BD Biosciences 553801 |
| **CD44** | Percp 5.5 | Rat IgG2b. κ | 1/600 | Biolegend BLE103032 |
| **CD103** | PE-Cya7 | Hamster IgG | 1/200 | Biolegend 121426 |
| **CD62L** | PE | Rat IgG2a. κ | 1/200 | BD Biosciences 553151 |
| **Siglec F** | PE-Texas Red | Rat IgG2a. κ | 1/250 | BD Biosciences 562757 |
| **CD8β** | APC | Rat IgG2a. κ | 1/200 | Biolegend 140410 |
| **CD3ε** | AF 700 | Rat IgG2b. κ | 1/100 | Biolegend BLE152316 |
| **CD8α** | APC-Cya7 | Rat IgG2a. κ | 1/400 | Biolegend BLE100714 |
| **CD16/30** | - | Rat IgG2b. κ | 1/150 | BD Biosciences 553142 |

*Supplementary Table S4: Antibodies used for CITE-seq analysis*

| **Target / marker** | **Clone** | **Isotype** | **Barcode sequence** | **Reference** |
| --- | --- | --- | --- | --- |
| **CD4** | RM4-5 | Rat IgG2a, κ | AACAAGACCCTTGAG | Biolegend 100569 |
| **CD8a** | 53-6.7 | Rat IgG2a, κ | TACCCGTAATAGCGT | Biolegend 100773 |
| **PD1** | RMP1-30 | Rat IgG2b, κ | GAAAGTCAAAGCACT | Biolegend 109123 |
| **LY6C** | HK1.4 | Rat IgG2c, κ | AAGTCGTGAGGCATG | Biolegend 128047 |
| **LY6G** | 1A8 | Rat IgG2a, κ | ACATTGACGCAACTA | Biolegend 127655 |
| **OX-2** | OX-90 | Rat IgG2a, κ | TCAATTCCGGTAGTC | Biolegend 123811 |
| **CD25** | PC61 | Rat IgG1, λ | ACCATGAGACACAGT | Biolegend 102055 |
| **CD115** | AFS98 | Rat IgG2a, κ | TTCCGTTGTTGTGAG | Biolegend 135533 |
| **CD11c** | N418 | Armenian Hamster IgG | GTTATGGACGCTTGC | Biolegend 117355 |
| **NK1.1** | PK136 | Mouse IgG2a, κ | GTAACATTACTCGTC | Biolegend 108755 |
| **OX-40** | OX-86 | Rat IgG1, κ | CTCACCTACCTATGG | Biolegend 119426 |
| **GITR** | DTA-1 | Rat IgG2b, κ | GGCACTCTGTAACAT | Biolegend 126319 |
| **CD103** | 2E7 | Armenian Hamster IgG | TTCATTAGCCCGCTG | Biolegend 121437 |
| **CD8b** | YTS156.7.7 | Rat IgG2b, κ | TTCCCTCTATGGAGC | Biolegend 126623 |
| **CD172a** | P84 | Rat IgG1, κ | GATTCCCTTGTAGCA | Biolegend 144033 |
| **CD163** | S15049I | Rat IgG2a, κ | GAGCAAGATTAAGAC | Biolegend 155303 |

**Supplementary legend figures**

**Supplementary Figure S1: Validation of intestine-specific FXR knockout mice. (a)** qPCR quantification of *Fxr* gene expression in intestine, liver and kidney from Ctrl-flox and ^int^FXR KO mice under standard diet (n = 4-6/genotype). **(b)** Representative full-length, unprocessed Western blot images and **(c)** quantification of FXR protein expression in colon and liver from Ctrl-flox and ^int^FXR KO mice under standard diet (n = 3-5/genotype). **(d-e)** qPCR quantification of the expression of FXR target genes in intestine **(d)** and liver **(e)** from Ctrl-flox and ^int^FXR KO mice under standard diet (n = 4-5/genotype). **(f)** Body weight evolution of Ctrl-flox and ^int^FXR KO mice under standard diet between 7 and 12 weeks of age (n = 11-12/genotype). **(g)** qPCR quantification of the expression of intestinal FXR target genes and **(h)** epithelial cell marker genes in Ctrl-flox and ^int^FXR KO mice after 24 weeks of HFSC diet (**g**: n = 12/genotype; **h**: n = 5-8/genotype). Data are presented as mean +/- SEM. Statistical analyses: For (**a–c**, **g** and **h**), Mann–Whitney tests: *p < 0.05, **p < 0.01, ***p < 0.001, ****p < 0.0001. For **(f)**, two-way ANOVA followed by Bonferroni post hoc test. Time effect: p < 0.001; no significant genotype effect.

(Abbreviations: Duo, duodenum; HFSC, high-fat, high-sucrose, high-cholesterol diet; Jej, jejunum; Lyz1, Lysozyme 1; Muc2, Mucin 2)

**Supplementary Figure S2: Impact of intestinal FXR-deficiency on intestinal immune cells**. **(a)** Gating strategy used for flow cytometry analysis of intestinal immune cell populations. **(b)** Quantification of immune cell populations and **(c)** T lymphocyte subpopulations isolated from the small intestinal epithelium of Ctrl-flox and ^int^FXR KO mice after 32 weeks of standard diet (n = 9-12/genotype). **(d)** Quantification of immune cell populations and **(e)** T lymphocyte subpopulations isolated from the small intestinal *lamina propria* of Ctrl-flox and ^int^FXR KO mice after 32 weeks of standard diet (**d**: n = 9-12/genotype; **e**: n = 8-12/genotype). **(f)** Quantification of immune cell populations and **(g)** T lymphocyte subpopulations isolated from the small intestinal *lamina propria* of Ctrl-flox and ^int^FXR KO mice after 24 weeks of HFSC diet (n = 9-10/genotype). Data are presented as mean +/- SEM. Statistical analysis: Mann–Whitney tests. **p < 0.05*, ***p < 0.01*.

(Abbreviations: B, B lymphocytes; DC, Dendritic cells; HFSC, High fat high sucrose high cholesterol; IEC, Intra-epithelial cells; MΦ, macrophages; SI, Small intestine; T, T lymphocytes)

**Supplementary Figure S3: Intestinal FXR deficiency does not alter hepatic parameters in standard diet-fed mice**. **(a)** Liver weight measured at sacrifice in Ctrl-flox and ^int^FXR KO mice under standard diet for 32 weeks (n = 10-12/genotype). **(b)**. Representative hematoxylin and eosin (H&E)-stained liver sections from Ctrl-flox and ^int^FXR KO mice after 32 weeks of standard diet (scale bar = 200 µm). **(c)** Hepatic cholesterol and **(d)** TG concentrations normalized to total hepatic protein content in Ctrl-flox and ^int^FXR KO mice under standard diet for 32 weeks (n = 8/genotype). **(e)** Quantification of inflammatory foci in liver sections from Ctrl-flox and ^int^FXR KO mice after 32 weeks of standard diet (n= 3/genotype). The average number of inflammatory foci was determined from 10 equivalent 20× fields per slide. **(f)** Blinded NAFLD Activity Score (NAS) assessment performed by an experienced liver pathologist in Ctrl-flox and ^int^FXR KO mice after 24 weeks of HFSC diet feeding (n = 12/genotype). Data are presented as mean +/- SEM. Statistical analysis: Mann–Whitney tests.

(Abbreviations: H&E, Hematoxylin and eosin; TG, Triglycerides)

**Supplementary Figure S4: Identification of hepatic DC, T lymphocyte, and neutrophil subclusters by scRNA-seq analysis. (a)** Heatmaps showing the top expressed genes for each immune cell cluster identified by scRNA-seq analysis in hepatic immune cells isolated from Ctrl-flox and ^int^FXR KO mice after 24 weeks of HFSC diet. **(b)** UMAP representation of cDC subclusters identified by scRNA-seq analysis and **(c)** quantification of the cDC1:cDC2 ratio in Ctrl-flox and ^int^FXR KO mice after 24 weeks of HFSC diet. Cell numbers were downsampled to the lowest number of cells per genotype (n = 128 cells). **(d)** UMAP representation of T-lymphocyte subclusters identified by scRNA-seq analysis and **(e)** quantification of the CD4:CD8 T ratio in Ctrl-flox and ^int^FXR KO mice after 24 weeks of HFSC diet. Cell numbers were downsampled to the lowest number of cells per genotype (n = 46 cells). **(f)** UMAP representation of neutrophil subclusters identified by scRNA-seq analysis in Ctrl-flox and ^int^FXR KO mice u after 24 weeks of HFSC diet. Cell numbers were downsampled to the lowest number of cells per genotype (n = 138 cells). **(g)** Relative proportions of neutrophil subtypes in Ctrl-flox and ^int^FXR KO mice after 24 weeks of HFSC diet. For all n= 3 mice / genotype. Data are presented as mean +/- SEM.

(Abbreviation: cDC, conventional dendritic cells; HFSC, High fat high sucrose high cholesterol; NK, Natural Killer; UMAP, Uniform Manifold Approximation and Projection).

**Supplementary figures**

*Supplementary Figure S1*

*Supplementary Figure S2*

*Supplementary Figure S3*

*Supplementary Figure S4*
